# Diagnostic value of procalcitonin and IL-6 for sepsis of older patients and other patients in the emergency department

**DOI:** 10.1186/cc14140

**Published:** 2015-03-16

**Authors:** SH Woo

**Affiliations:** 1 College of Medicine, The Catholic University of Korea, Incheon St. Mary's Hospital, Incheon, South Korea

## Introduction

Emergency physicians are able to identify severely ill older patients with SIRS in the emergency department (ED). A previous study showed that elevated procalcitonin (PCT) and IL-6 level is a known marker of sepsis and predicts bacteremia and mortality. We assessed the diagnostic value of PCT and IL-6 in older patients and other patients with SIRS and sepsis in the ED.

## Methods

This retrospective cohort study was conducted from January 2013 to December 2013. We enrolled 122 patients with SIRS, 55 were classified as the older age group (>65 years of age). Measurement of serum PCT, IL-6, and white blood cell count was performed on initial admission to the ED. We analyzed these markers in older patients and other patients groups with sepsis.

## Results

Of the 55 patients in the older group 33 (60%) patients had sepsis, and 40 (59.7%) patients of the other group had sepsis. PCT and IL-6 levels were significantly higher in other patients with sepsis (*P *< 0.001, *P *< 0.001). But PCT and IL-6 levels were not higher in old age patients with sepsis (*P *= 0.400, *P *= 0.169). The area under the receiver operating characteristic curve (AUC) for diagnosis of sepsis according to PCT and IL-6 was 0.823, and 0.772 for the other patients group. The diagnostic sensitivity, specificity, positive predictive value, and negative predictive value of PCT for sepsis in other patients group were 79.5%, 81.5%, 86.1%, and 73.3% respectively, with a PCT cutoff value of 0.18 ng/ml. The diagnostic sensitivity, specificity, positive predictive value, and negative predictive value of IL-6 for sepsis in other patients group were 67.5%, 81.5%, 84.4%, and 62.9% respectively, with a IL-6 cutoff value of 74.43 pg/ml.

## Conclusion

PCT and IL-6 is useful predictive markers for diagnosing sepsis in adult patients (<65 years of age) with SIRS in the ED. But these markers are not useful for identification of sepsis in older patients.

